# Collaborative Care from the Family Perspective

**DOI:** 10.1192/j.eurpsy.2025.61

**Published:** 2025-08-26

**Authors:** J. Saunders

**Affiliations:** EUFAMI, Leuven, Belgium

## Abstract

**Abstract:**

This Symposium will consider the process of collaboration from the family persective.

EUFAMI is of the view that the involvement of families in the process of care and recovery is accepted in theory but is not sufficiently practiced. concerned and involved family members are often excluded from important decisions that affect their loved ones and ultimately affect the quality of care and recvovery.

There will be a brief introduction from a representative of EUFAMI followed by a presentarion from a family member. Issues such as involvement in earlt indentivication and assessment,diagnosis, planning of interventions and supporting the recovery process will be considered.Issues such as the rights of famililies, concerns abour relapss, patiient confidentiality and the sharing of infromaton will all be considered in the context of the delivery of mentsl helth care services in the community

**Disclosure of Interest:**

None Declared

